# Improving Causality Induction with Category Learning

**DOI:** 10.1155/2014/650147

**Published:** 2014-04-30

**Authors:** Yi Guo, Zhihong Wang, Zhiqing Shao

**Affiliations:** ^1^Department of Computer Science and Engineering, East China University of Science and Technology, P.O. Box 408, Shanghai 200237, China; ^2^School of Information Science and Technology, Shihezi University, Shihezi 832003, China

## Abstract

Causal relations are of fundamental importance for human perception and reasoning. According to the nature of causality, causality has explicit and implicit forms. In the case of explicit form, causal-effect relations exist at either clausal or discourse levels. The implicit causal-effect relations heavily rely on empirical analysis and evidence accumulation. This paper proposes a comprehensive causality extraction system (CL-CIS) integrated with the means of category-learning. CL-CIS considers cause-effect relations in both explicit and implicit forms and especially practices the relation between category and causality in computation. In elaborately designed experiments, CL-CIS is evaluated together with general causality analysis system (GCAS) and general causality analysis system with learning (GCAS-L), and it testified to its own capability and performance in construction of cause-effect relations. This paper confirms the expectation that the precision and coverage of causality induction can be remarkably improved by means of causal and category learning.

## 1. Introduction

A general philosophical definition of causality states that, the philosophical concept of causality refers to the set of all particular causal or cause-and-effect relations [1]. Causal relations are of fundamental importance for human perception and reasoning. Since ignoring causal relationships may have fatal consequences, their knowledge plays a crucial role in daily life to ensure survival in an ever changing environment.

In many research works, the causality generally refers to the existence of causality in mathematics and physics. Causalities are often investigated in situations influenced by uncertainty involving several variables. Thus, causalities, which can be presented in terms of flows among processes or events, are generally expressed in mathematical languages and analyzed in a mathematical manner. Therefore, statistics and probability theories seem to be the most popular mathematical languages for modeling causality in most scientific disciplines. There is an extensive range of literature on causality modeling, applying and combining mathematical logic, graph theory, Markov models, Bayesian probability, and so forth [2]. But, they seem not to be able to predominate all relevant issues or questions.

In recent years, clarification and extraction of cause-effect relationships among texts (e.g., objects or events), causality extraction, is elevated to a prominent research topic in text mining, knowledge engineering, and knowledge management.

A variety of research works testify that causalities in texts can be extracted at three technology levels. The first is the clausal level (CL), which includes cue phrases and lexical clues [3] and semantic similarity [4]. The second is the discourse level (DL), which implements connective markers [5] and constructs discourse relations [6]. The third is the mode level (ML), which extracts causal relations from a QA system [7, 8], applies commonsense rules [9], associative memory [10], and Chain Event Graphs [11].

According to the nature of causality in texts, causality has explicit and implicit forms. In the case of explicit form, causal-effect relations exist at either clausal or discourse levels. The implicit causal-effect relations heavily rely on empirical analysis and evidence accumulation.

Moreover, Waldmann and Hagmayer [12], in their research work of cognitive psychology, state that “categories that have been acquired in previous learning contexts may influence subsequent causal learning,” which indicate that (1) the category information about objects or events in texts is a necessary supplement for causality extraction, and (2) the category information has impact on subsequent learning-based cause-effect identification.

Based on the facts, the task of causality extraction, even induction, in texts could not be accomplished in an arbitrary manner. This paper proposes a comprehensive causality extraction system (CL-CIS) integrated with the means of category-learning. CL-CIS considers cause-effect relations in both explicit and implicit forms and especially practices the relation between category and causality in computation.

The rest of this paper is organized as follows.  Section 2 states the causality, category information, and the relationships between them in texts and constructs the theoretical foundation of our research work.  Section 3 expatiates the methodology and the technical details of system structure and kernel algorithms.  Section 4 focuses on experiment illustration and result analysis of the experimental results.  Section 5 concludes this paper and provides future research works.

## 2. Causality and Category

Traditionally, research about the representation of causal relations and research about the representation of categories were separated. This research strategy rests on the assumption that categories summarize objects or events on the basis of their similarity structure, whereas causality refers to relations between causal objects or events. Literature [12] proves that the relationship between causality and categorization is more dynamic than previously thought.

### 2.1. Causality

The standard view guiding research on causality presupposes the existence of objective networks of causes and effects that cognitive systems try to mirror. Regardless of whether causal learning is viewed as the attempt to induce causality on the basis of statistical information or on the basis of mechanism information, it is generally assumed that the goal of causal learning is to form adequate representations of the texture of the causal world.

### 2.2. Causality Rests on Fixed Categories

Studies on causal learning typically investigate trial-by-trial learning tasks which involve learning the contingencies between causes and effects. In a large number of studies which focus on causal contingency learning. A characteristic feature of these tasks is that they present categorized events representing causes and effects which are statistically related. Cause and effect categories are viewed as fixed entities that are already present prior to the learning task. The goal of learning is to estimate causal strength of individual causal links or to induce causal models on the basis of observed covariations. The role of cause and effect categories in the learning process is not the focus of interest in these approaches; they are simply viewed as given.

A similar approach underlies research on the relationship between categories and causality. According to the view that categorization is theory-based, traditional similarity-based accounts of categorization are deficient because they ignore the fact that many categories are grounded in knowledge about causal structures [13]. In natural concepts, features often represent causes or effects with the category label referring to a complex causal model. For example, disease categories frequently refer to common-cause models of diseases with the category features representing causes (e.g., virus) and effects (e.g., symptoms) within this causal model. A number of studies using these and similar materials have shown that the type of causal model connecting otherwise identical cause and effect features influences learning, typicality judgments, or generalization [14–17]. The main goal of these studies was to investigate the effect of different causal relations connecting the causal features. As in contingency learning studies, the cause and effect features within the causal models were treated as fixed, categorized entities, which already existed prior to the learning context.

### 2.3. Categories Shape Causality

It is certainly true that many interesting insights can be gained from investigating how people learn about causal models on the basis of preexisting cause and effect categories. However, there is also a link between categories and causality in the opposite direction. The categories that have been acquired in previous learning contexts may have a crucial influence on subsequent causal learning.

The basis of the potential influence of categories on causal induction lies in the fact that the acquisition and use of causal knowledge is based on categorized events. Causal laws, such as the fact that smoking causes heart disease, can only be noticed on the basis of events that are categorized (e.g., events of smoking and cases of heart disease).

Without such categories causal laws neither could be detected nor could causal knowledge be applied to new cases. Thus, causal knowledge not only affects the creation of categories, it also presupposes already existing categories for the description of causes and effects. The potential influence of categories is due to the fact that one of the most important cues to causality is statistical covariation between causes and effects.

**Figure 1:**
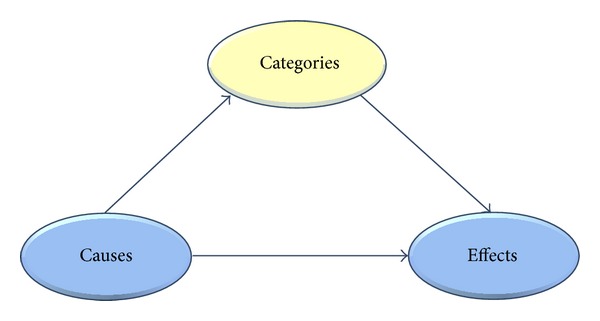
Possible routes of category learning between causes and effects.

To study the relation between categories and causal induction, [12] have developed a new paradigm that consists of three phases, the category learning phase, the causal learning phase, and the third test phase. The main goal is to answer the question that under what condition learners will tend to activate the categorical information (Figure 1) from the earlier category learning phase when learning about causal contingencies in the later phase. This effect is entailed by the fact that the alternative categories form different reference classes.

In category learning phase, causes will be classified into the distinct categories (upper left arrow in Figure 1). In causal learning phase, causes in collection are paired with the presence or absence of an effect (lower arrow). In the subsequent test phase, the test causes are rated with the likelihood of the effect. The crucial question is when people would go through the upper route in Figure 1 and assign the test causes to the categories in the category learning phase or whether they would stick to the lower route and induce new categories within causal learning phase.

If the learning strategy opted for the lower route, one possible solution may be to induce new categories that are maximally predictive of the effects. This solution would obviously generate maximally predictive categories. Lien and Cheng [18] reported a research that is consistent with the maximal-contrast hypothesis. Their experiments show that the substances are categorized according to the feature and to the hierarchical level that were maximally predictive for the effect. Thus, the induced substance category was determined by its suitability for predicting the effect. Lien and Cheng [18] interpreted this as evidence for their maximal-contrast hypothesis. In another word, people tend to induce categories that maximize their causal predictability.

**Figure 2:**
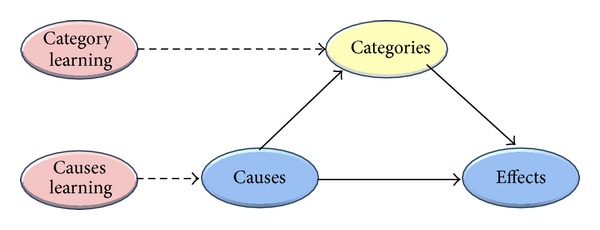
Enhanced strategy of category learning between causes and effects.

In sum, our research addresses the question of which route learners will go. Will they routinely go through the upper road and activate category knowledge when learning about novel effects? A simple connectionist one-layer network that may be used to understand the task we are going to explore in our experiments, or will they go the lower road and learn a new set of categories on the basis of causal information in causal learning phase that is maximally predictive within this learning phase? Thus, Figure 1 is enhanced with two learning phases as shown in Figure 2.

## 3. Research Methodology and System Structure

### 3.1. Research Methodology

Causality is a fundamental concept in human thinking and reasoning. It is not surprising that most, if not all, languages in the world have a range of lexical expressions specifically designed for communicating causal relations.

This paper focuses on explicit causality markers and implicit causal relations in text. The explicit causality markers include two grammatically different types of causality markers in English. We investigate the semantic contrasts expressed by different causal auxiliary verbs, marking causal relations expressed* within* one clause, and those expressed by different causal connectives, marking causal relations* between* clauses.

#### 3.1.1. Causality in Verbs

Some instances of causality in verbs are listed below. These verbs include, but are not limited to: “make,” “let,” “have,” “cause,” and their synonyms from WordNet [19] which is generally referred to as an online lexical database.

 
*[The extreme cold] cause made/caused even [the rivers (to) freeze] effect.*
 
*[She] cause made/had [her son empty his plate] effect, despite his complaints.*

#### 3.1.2. Causality in Discourse Connectives

For a complex sentence, the predicative or relative clauses of the noun synonyms of “cause” are labeled as “potential cause” of antecedent sentence or main clause. Additionally, each clause inducted with “since,” “as,” or “because” is also labeled as “potential cause” of its main clause, which is labeled as “potential effect.”

Table 1 lists the statistics for above backward causality collected upon Reuters-21578, currently the most widely used test collection for text processing research, and BBC-News2000, collected by a self-developed Web Crawler from http://www.bbc.co.uk/. The forward causality, where in presentation order the cause precedes the effect, is the most frequently used ones, for example, “therefore,” “because of that,” “that is why,” and “so.” As the forward causality connectives are one hundred percent strong causality indicators, the backward causality connectives in Table 1 need more specific notation. For example, in all “because” connectives in collections, 83% of them indicate objective reasons, while the other 17% indicate subjective reasons. Meanwhile, the “because” connectives persist 50% of all the connectives including “because,” “for,” “as,” “since,” “while,” and other miscellaneous connectives. The “for,” “as,” “since,” “while,” and other miscellaneous connectives correspondingly hold 18%, 13%, 6%, 6%, and 7% of all the connectives in collections.

#### 3.1.3. Implicit Causality in Texts

Causal effect relations are general connections in the objective world, and the causality sentences exist in languages in a pervasive manner. The explicit causality sentences are those conducted by backward and forward causality connectives listed in Section 3.1.2, while the implicit causality sentences are those connected with other connectives, even without any one. Our research works have testified that there are five types of implicit causality sentences shown in Table 2.

In the practical English texts, there exist a few interpersonal verbs like “praise” and “apologize,” thus supplying information about whose behavior or state is the more likely immediate cause of the event at hand. Because it is conveyed implicitly as part of the meaning of the verb, this probabilistic cue is usually referred to as implicit causality.

Such exemplar implicit causality verbs [20] adopted in this paper are listed in Table 3 together with their bias indicating the probabilities as causal cues; for example, 1.00 is the causal baseline; the higher the bias value is, the more likely is the cause.

### 3.2. System Description

**Figure 3:**
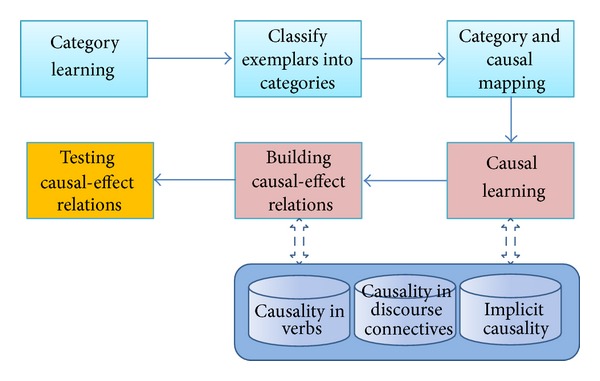
System framework of CL-CIS.

Our causality induction system with assistance of category learning, named CL-CIS, is composed with the following functional modules (shown in Figure 3): category learning, classify exemplars into categories, category and causal mapping, causal learning, building causal-effect relations, and the final testing module. CL-CIS also includes three reference libraries (databases): causality in verbs, causality in discourse connectives, and implicit causality for query and assistance.  Table 4 compares the composition difference among general causality analysis system (GCAS), general causality analysis system with learning (GCAS-L), and CL-CIS. As the construction of three libraries is elaborated in Section 3.1 Methodology, this section concentrates on the six functional modules.

#### 3.2.1. Category Learning (CL)

The category learning module builds up different distinct and exhaustive categories according to semantic contents (e.g., noun phrases, verb phrases) of sentences in our text collections. This module is a basic preprocessing step and the target is to construct a collection of distinct and exhaustive categories with the text learning methods.

#### 3.2.2. Classify Exemplars into Categories (CEC)

In the classify exemplars into categories module, each sentence is treated as an individual independent event and parsed into phrases. The classification is based on the comparison of a sentence with features of each category, so as to set up the category background knowledge for each sentence. For example, the concept* bank* can be clarified as a finance organization or a river body boundary with corresponding category background knowledge of its sentence and contexts.

#### 3.2.3. Category and Causal Mapping (CCM)

This module constructs the category-causal-effect mapping relations; for example, a virus from a predefined category causes specific disease-related symptoms, such as a swelling of the spleen (splenomegaly). The construction mechanism, to a great extent, is based on collection, storage, and indexing of massive cases.

#### 3.2.4. Causal Learning (CauL)

As analyzed before, a simple connectionist one-layer network that may be used to understand the task of when to activate category knowledge when learning about novel effects. This module adopts a simple recurrent neural network (SRNN) to simulate the connectionist model.

**Figure 4:**
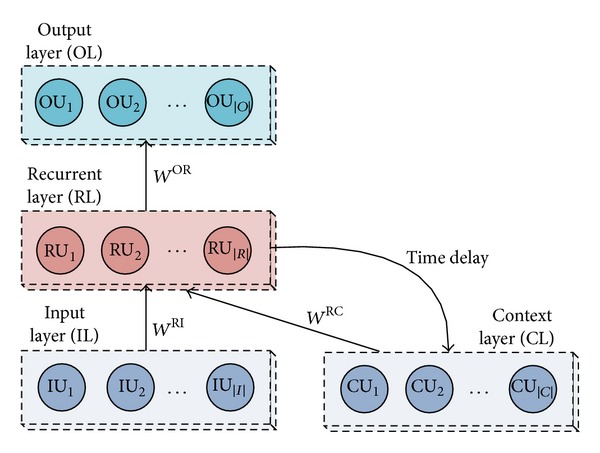
Architecture of SRNN.

Symbols in Figure 4 are defined in Table 5: the first order weight matrices **W** ^**RI**^ and **W** ^**OR**^ fully connect the units of the input layer (IL), the recurrent layer (RL), and the output layer (OL), respectively, as in the feed forward multilayer perceptron (MLP). The current activities of recurrent units RU^(*t*)^ are fed back through time delay connections to the context layer, which is presented as CU^(*t*+1)^ = RU^(*t*)^.

Therefore, each unit in recurrent layer is fed by activities of all recurrent units from previous time step through recurrent weight matrix **W** ^**RC**^. The context layer, which is composed of activities of recurrent units from previous time step, can be viewed as an extension of input layer to the recurrent layer. Above working procedure represents the memory of the network via holding contextual information from previous time steps.

The weight matrices *W* ^RI^, *W* ^RC^, and *W* ^OR^ are presented as follows:

(1) WRI=[(w1RI)T,(w2RI)T,…,(w|R|RI)T]=[w11
ri
w12
ri
⋯w1,|R|
ri
w21
ri
w22
ri
⋯w1,|R|
ri
⋮⋮⋱⋮w|I|,1
ri
w|I|,2
ri
⋯w|I|,|R|
ri
],WRC=[(w1RC)T,(w2RC)T,…,(w|R|RC)T]=[w11rcw12rc⋯w1,|R|rcw21rcw22rc⋯w1,|R|rc⋮⋮⋱⋮w|C|,1rcw|C|,2rc⋯w|C|,|R|rc],WOR=[(w1OR)T,(w2OR)T,…,(w|O|OR)T]=[w11orw12or⋯w1,|O|orw21orw22or⋯w1,|O|or⋮⋮⋱⋮w|R|,1orw|R|,2or⋯w|R|,|O|or].

In the above formulations, (*w*_*k*_^RI^)^*T*^ is the transpose of *w*_*k*_^RI^ for the instance of *W* ^RI^, where *w*_*k*_^RI^ is a row vector and (*w*_*k*_^RI^)^*T*^ is the column vector of the same elements. The vector *w*_*k*_^RI^ = (*w*_1*k*_^ ri ^, *w*_2*k*_^ ri ^,…, *w*_|*I*|,*k*_^ ri ^) represents the weights from all the input layer units to the recurrent (hidden) layer unit RU_*k*_. The same conclusion applies with *W* ^RC^ and *W* ^OR^.

Given an input pattern in time **t**, **I** **U** ^(**t**)^ = (IU_1_^(*t*)^, IU_2_^(*t*)^,…, IU_|*I*|_^(*t*)^), and recurrent activities **R** **U** ^(**t**)^ = (RU_1_^(*t*)^, RU_2_^(*t*)^,…, RU_|*R*|_^(*t*)^) for the *i*th recurrent unit, the net input RU_*i*_^′(*t*)^ and output activity RU_*i*_^(*t*)^ are calculated as follows:

(2) RUi′(t)=IU(t)·(wiRI)T+RU(t−1)·(wiRC)T=∑j=1|I|IUj(t)wji
ri
+∑j=1|R|RUj(t−1)wjirc,RUi(t)=f(RUi′(t)).

For the* k*th output unit, its net input OU_*k*_^′(*t*)^ and output activity OU_*k*_^(*t*)^ are calculated as (3). Consider the following:

(3) OUk′(t)=RU(t)·(wkOR)T=∑j=1|R|RUj(t)wjkor,OUk(t)=f(OUk′(t)).

Here, the activation function *f* applies the logistic sigmoid function (4) in this paper. Consider the following:

(4) $$\begin{array}{c} f\left(x\right)=\frac{1}{1+e^{-x}}=\frac{e^{x}}{1+e^{x}}. \end{array}$$

#### 3.2.5. Building Causal-Effect Relations (BCER)

This module builds up the “cause-effect” pairs and connections with the inputs of SRNN and corresponding outputs. The causality detection results could include four possible types: (1) single cause-effect pairs in which any two pairs are independent from each other; (2) cause-effect chains, which are formed with more than one cause-effect pairs connected together (an effect is a cause of another effect); (3) one-cause-multiple-effect pairs; (4) multiple-cause-one-effect pairs. All of the causality connections are archived in a database. Both CauL and BCER modules exploit three libraries (databases): causality in verbs, causality in discourse connectives, and implicit causality, for reference.

#### 3.2.6. Testing Causal-Effect Relations (TCER)

This module tests and verifies a new processed causal-effect relation with our existing and expanding collection of massive causal-effect relations. The concrete technology includes comparison of triples 〈Category, Cause, Effect〉.

## 4. Experiments and Results

Our experiments test general causality analysis system (GCAS), general causality analysis system with learning (GCAS-L), and CL-CIS together, in order to examine and reveal the assertion that category information is a necessary supplement for causality extraction and has impact on subsequent learning-based cause-effect identification.

In our experiments, we have used two text collections: (1) Reuters-21578, currently the most widely used test collection for text processing research; (2) BBC-News2000, collected by a self-developed Web Crawler from http://www.bbc.co.uk/.

BBC-News2000 collected 2000 news articles which have been pruned off unnecessary Web page elements, such as html tags, images, URLs, and so forth. BBC-News2000 is not categorized and is treated as a whole hybrid.

30 judges are involved to manually search cause-effect pairs and construct standard causality references (SCR), SCR-Reuters for Reuters-21578, and SCR-BBC for BBC-News2000. Due to limited human resources, in current stage, SCR-Reuters only covers 2000 documents,* newid* ranges from 1 to 2000, contained in files “reut2-000.sgm” and “reut2-001.sgm.” The rest of the files of Reuters-21578 are still involved in the training phase.

**Figure 5:**
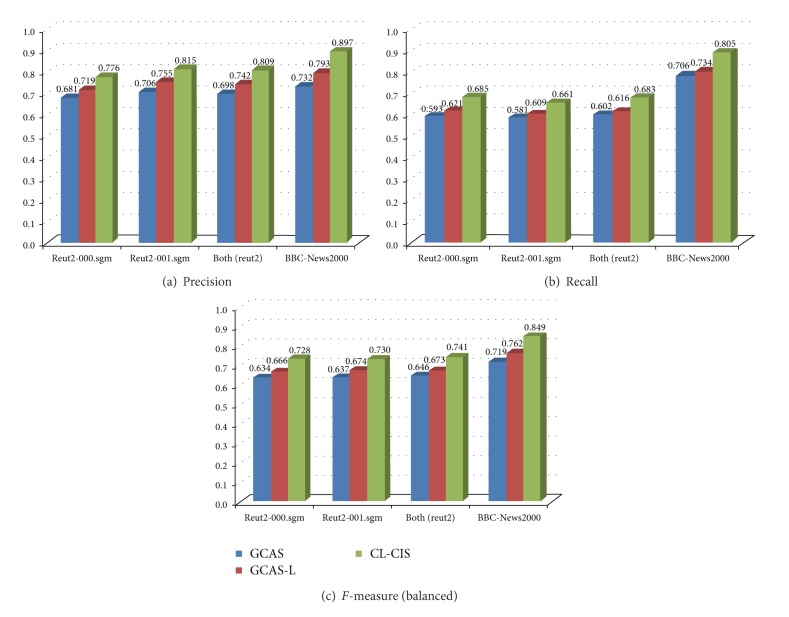
Evaluation results of GCAS, GCAS-L, and CL-CIS.

The performances of GCAS, GCAS-L, and CL-CIS are evaluated with precision, recall, and* F*-measure [21], the traditional measures that have been widely applied by most information retrieval systems to analyze and evaluate their performance. The* F*-measure is a harmonic combination of the precision and recall values used in information retrieval. As shown in Tables 6, 7, and 8, the experimental results state that (1) GCAS scores from 0.681 to 0.732 on precision and from 0.581 to 0.706 on recall; (2) GCAS-L scores from 0.719 to 0.793 on precision and from 0.609 to 0.734 on recall; (3) CL-CIS scores from 0.776 to 0.897 on precision and from 0.661 to 0.805 on recall.  Figure 5 explicitly states that (1) the general causality analysis system with learning (GCAS-L) performances better than the general causality analysis system (GCAS) on all evaluation measures; (2) the causality analysis system strengthened with causal and category learning (CL-CIS) exceeds GCAS-L in the meantime.

## 5. Concluding Remarks

In recent years, detection and clarification of cause-effect relationships among texts, events, or objects has been elevated to a prominent research topic of natural and social sciences over the human knowledge development history.

This paper demonstrates a novel comprehensive causality extraction system (CL-CIS) integrated with the means of category-learning. CL-CIS considers cause-effect relations in both explicit and implicit forms and especially practices the relation between category and causality in computation.

CL-CIS is inspired with cognitive philosophy in category and causality. In causality extraction and induction tasks, CL-CIS implements a simple recurrent neural network (SRNN) to simulate human associative memory, which has the ability to associate different types of inputs when processing information.

In elaborately designed experimental tasks, CL-CIS has been examined in full with two text collections, Reuters-21578 and BBC-News2000. The experimental results have testified the capability and performance of CL-CIS in construction of cause-effect relations and also confirmed the expectation that the means of causal and category learning will improve the precision and coverage of causality induction in a notable manner.

## Figures and Tables

**Table 1:** Statistics for backward causality connectives.

Connectives	For objective reason	For subjective reason	Total percentage in all connectives
Because	83%	17%	50%
For	29%	71%	18%
As	60%	40%	13%
Since	14%	86%	6%
While	14%	86%	6%
*Misc *	0%	100%	7%

Total	56%	44%	100%

**Table 2:** Types of implicit causality sentences.

Types	Subtypes	Exemplar sentences
Compound sentences	Cause-effect sentences connected with “and”	Cause-effect: (1) This was the first time I made an international call, and my heart was beating fast. (2) The filter is much more efficient than the primary filter and it removes all remaining solid particles from the fuel.
		Effect-cause: (3) The crops had failed, and there had not been any rain for months. (4) Aluminum is used as engineering material for planes and spaceships and it is light and tough.
	Cause-effect sentences without connectives	Effect-cause: (5) My heart sank. Some of them did not weigh more than 135 pounds, and the others looked too young to be in trouble. (6) …but the Voyager's captain and three crewmen had stayed in board. They had hoped the storm would die out and they could save the ship.
		Cause-effect: (7) The red distress flare shot up from the sinking ship, the Voyager. Everyman aboard our Coast Guard cutter knew that time had run out.

Relative clauses	SV, SVO, SVC, SVOC, SVOO, SVA, SVOA	(8) To make an atom we have to use uranium, in which the atoms are available for fission. (9) We know that a cat, whose eyes can take in more rays of light than our eyes, can see clearly in the night.

***If*** clauses		(10) This system of subsidies must be maintained if the farmer will suffer considerable losses if it is abolished. (11) If the water will rise above this level, we must warn everybody in the neighborhood.

***That*** clauses		(12) The act was even the bolder that he stood utterly alone. (13) The crying was all the more racking that she was not hysterical but hopeless.

SVO-SVOC		(14) Her falling ill spoiled everything. (15) Timidity and shamefacedness caused her to stand back and looked indifferently away.

**Table 3:** Exemplar implicit causality verbs.

NP1 verbs	Bias	NP2 verbs	Bias
*Amazed *	1.19	*Admire *	2.00
*Annoy *	1.19	*Adore *	1.86
*Apologize *	1.00	*Appreciate *	2.00
*Be in the way *	1.08	*Comfort *	1.91
*Beg *	1.17	*Compliment on something *	1.96
*Bore *	1.05	*Congratulate *	1.95
*Call *	1.19	*Criticize *	2.00
*Confess *	1.03	*Envy *	2.00
*Disappoint *	1.03	*Fear *	2.00
*Disturb *	1.14	*Fire *	1.96
*Fascinate *	1.00	*Hate *	1.96
*Hurt *	1.13	*Hold in contempt *	2.00
*Inspire *	1.23	*Hold responsible *	1.91
*Intimidate *	1.23	*Loathe *	1.86
*Irritate *	1.22	*Love *	1.86
*Lie to *	1.22	*Praise *	1.96
*Mislead *	1.22	*Press charges against *	1.91
*Swindle *	1.13	*Punish *	1.95
*Win *	1.19	*Respect *	1.95
*Worry *	1.19	*Thank *	1.82

**Table 4:** System composition comparison of GCAS, GCAS-L, and CL-CIS.

System composition	GCAS	GCAS-L	CL-CIS
Modules			
Category learning			***√***
Classify exemplars into categories			***√***
Category and causal mapping			***√***
Causal learning		***√***	***√***
Building causal-effect relations	***√***	***√***	***√***
Testing causal-effect relations	***√***	***√***	***√***
Libraries			
Causality in verbs	***√***	***√***	***√***
Causality in discourse connectives	*Optional *	***√***	***√***
Implicit causality		*Optional *	***√***

**Table 5:** Definition of SRNN Symbols.

Symbols	Definition
IU	A unit of input layer
RU	A unit of recurrent layer
CU	A unit of context layer
OU	A unit of output layer
|*I*|	The number of units in IL
|*R*|	The number of units in RL
|*C*|	The number of units in CL
|*O*|	The number of units in OL
**W** ^**RI**^	The weight vector from IL to RL
**W** ^**RC**^	The weight vector from CL to RL
**W** ^**OR**^	The weight vector from RL to OL

**Table 6:** Experimental results of GCAS.

Experiment files	Precision	Recall	*F*-measure
reut2-000.sgm (newid: 1–1000)	0.681	0.593	0.634
reut2-001.sgm (newid: 1001–2000)	0.706	0.581	0.637
Both (reut2) (newid: 1–2000)	0.698	0.602	0.646
BBC-News2000	0.732	0.706	0.719

**Table 7:** Experimental results of GCAS-L.

Experiment files	Precision	Recall	*F*-measure
reut2-000.sgm (newid: 1–1000)	0.719	0.621	0.666
reut2-001.sgm (newid: 1001–2000)	0.755	0.609	0.674
Both (reut2) (newid: 1–2000)	0.742	0.616	0.673
BBC-News2000	0.793	0.734	0.762

**Table 8:** Experimental results of CL-CIS.

Experiment files	Precision	Recall	*F*-measure
reut2-000.sgm (newid: 1–1000)	0.776	0.685	0.728
reut2-001.sgm (newid: 1001–2000)	0.815	0.661	0.730
Both (reut2) (newid: 1–2000)	0.809	0.683	0.741
BBC-News2000	0.897	0.805	0.849
